# Robotic-assisted totally transhiatal lymphadenectomy in the middle mediastinum for esophageal cancer

**DOI:** 10.1007/s11701-013-0398-z

**Published:** 2013-03-15

**Authors:** Kazuhiko Mori, Yukinori Yamagata, Ikuo Wada, Nobuyuki Shimizu, Sachiyo Nomura, Yasuyuki Seto

**Affiliations:** 1 Department of Gastrointestinal Surgery, Graduate School of Medicine, The University of Tokyo, Tokyo, Japan; 2 Department of Gastrointestinal Surgery, University of Tokyo, Hongo 7-3-1, Bunkyo-ku, Tokyo, 113-8655 Japan

**Keywords:** Robotics, Esophageal surgery, Minimally invasive surgery, Mediastinal lymph dissection, Transhiatal

## Abstract

Robotic-assisted esophagectomy has been introduced as a minimally invasive esophagectomy; however, transhiatal esophagectomy with extended lymphadenectomy in the middle retromediastinal field has never been reported so far. We have developed a totally transhiatal robotic manipulation which enables middle retromediastinal lymph dissection. With this method, transthoracic approach, which is associated with risk of lung injury and loss of ventilatory function, can be avoided in radical esophageal cancer surgery. The robotic arms and camera entered the mediastinum passing the hiatus, and the dissection was performed along the medial aspect of the mediastinal pleura, the pericardia, and the aorta. The devices were able to reach the middle mediastinum, and lymph nodes on the bilateral main bronchi and the carina were retrieved by robotic manipulation. We present here our first experience of laparoscopic transhiatal lymphadenectomy of the middle mediastinal field, including a video, which could not have been possible without robotic-assisted surgery.

**Electronic supplementary material:**

The online version of this article (doi:10.1007/s11701-013-0398-z) contains supplementary material, which is available to authorized users.

## Introduction

Minimally invasive esophagectomy (MIE) has been attempted by video-assisted thoracoscopic surgery with or without robotic device [1, 2]; however, MIE with radical lymphadenectomy usually includes a transthoracic procedure. Transthoracic, typically right-side, manipulation mandates one-lung ventilation and destruction of the thoracic wall and mediastinal pleura. One-lung ventilation is reported to result in mechanical damage to both the ventilated and collapsed lung [3, 4]. Because the esophagus and regional lymph nodes are located inside the bilateral mediastinal pleura, the radical esophagectomy can be theoretically completed without any damage to the thoracic wall, pleura, or lung. Therefore, totally transhiatal radical lymphadenectomy may well be carried out without the above-mentioned damage. We applied a robotic-assisted approach, which would be suitable for dissections in a narrow operative field, for transhiatal radical lymphadenectomy in the middle mediastinal field for esophageal cancer. Here we report our first experience of such robotic-assisted transhiatal radical esophagectomy with a video.

## Surgical technique

**Fig. 1:**
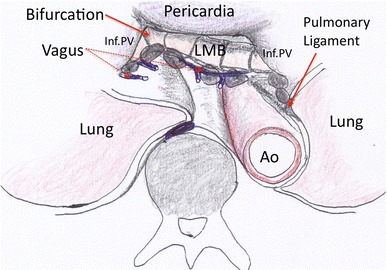
Mediastinal operative field observed by da Vinci S scope after resection of the esophagus and middle mediastinal node. Upper retromediastinal field could be observed beyond the middle mediastinal structures. *LMB* left main bronchus, *Ao* aorta, *inf. PV* inferior pulmonary vein

The patient was placed supine under epidural and general anesthesia throughout the operation. Prior to the abdominal procedures, a cervical procedure was performed through a cervical collar incision. The cervical procedure included regional lymph dissection in the upper mediastinum and dissection of the esophagus from the trachea and the bilateral recurrent laryngeal nerve. Subsequently, a small (6-cm) incision for minilaparotomy and five small incisions for the port sites were made in the epigastric area, and da Vinci S (Intuitive Surgical, Sunnyvale, CA) was applied for the following procedures. A Lap Disc (Ethicon Endosurgery Inc., Cincinnati, OH) was applied to maintain pneumoperitoneum. The operative field was obtained by retraction of the lateral segment of the left liver lobe by a retractor inserted through the Lap Disc. Three robotic arms and one assistant’s device entered the operative field via the port sites. Firstly, the esophagophrenic ligament was incised at the anterior aspect of the esophagogastric junction, and the mediastinum was entered through the phrenic crus. Lymph nodes along the middle and lower esophagus were dissected from the pericardia, the bilateral pleura, and the aorta by sharp dissection, mainly using monopolar scissors. As the dissection proceeded craniad, the roots of the bilateral inferior pulmonary veins were exposed. Thereafter, vagal fibers coming bilaterally from the main bronchi were visualized, and sharp dissection of these fibers enabled harvesting of lymph nodes in the pulmonary ligament, subcarinal, and bilateral main bronchial regions (Fig. 1; Video). Completion of these lymph dissections in the middle mediastinal field opened access to the cervical dissection field. With the dissection from the cervical procedure via the collar incision prior to the da Vinci manipulation, the whole esophagus could be freed from adhesions and attachments. The gastric pull-up was constructed by the following conventional open surgery after extending the epigastric incision, and the esophagus was harvested from the neck incision by transecting oral margin. A handsewn anastomosis between the remnant esophagus and the gastric pull-up was performed in the neck operative field.

## Results

The total operation time was 561 min, and the amount of blood loss was 720 ml. The detailed information is listed in Table 1. The patient was extubated soon after the operation, and oxygen inhalation was discontinued at the 4th postoperative day. The number of lymph nodes retrieved from the middle mediastinal field was 19, while the average number of nodes retrieved from the corresponding stations had been 15.1 in the latest 12 cases of conventional operation by the same surgical team. Minor anastomotic leakage occurred and needed 2 weeks’ restriction of food intake.

**Table 1:** Data profile of the operation

Patient	64-year-old male
Total time (min)	561
Cervical and laparoscopic phase (min)	171
da Vinci S operation (min)	245
Cervical anastomosis and other (min)	145
Blood loss	720 ml
Pathological findings	
Tumor stage (AJCC)	T1bN0M0
Histology	Squamous cell carcinoma
Number of harvested nodes	
Total	40
Right subbronchial	4
Left subbronchial	3
Subcarina	12
Postoperative hospital stay	29 days

*AJCC* America Joint Committee on Cancer

## Discussion

In surgical treatment of esophageal cancer, greater extent of lymphadenectomy is reported to be associated with increased survival [8]. Conventional transhiatal esophagectomy is recognized as a less invasive surgery but not oncologically equivalent to radical esophagectomy. Transhiatal dissection of the lower mediastinal field is possible with conventional endoscopic devices. However, as the operative field proceeds craniad, the motions of conventional devices suffer from increasing limitations and the right- and left-hand devices become parallel to one another. Thus, the lymph nodes in the middle mediastinum, if reached and visualized, are not available when using conventional laparoscopic devices. Meanwhile, the robot has arms with seven degrees of freedom and can move in a narrow cavity such as the mediastinum. We have routinely performed upper mediastinal lymph dissection through the cervical incision as far as the lower aspect of the aortic arch. Combining the middle mediastinal dissection by robotic approach, radical esophagectomy together with three-field lymphadenectomy could be completed without any transthoracic procedure.

Robotic-assisted surgeries are being reported in various surgical fields; however, no significant superiority as a minimally invasive surgery has been fully demonstrated in gastrointestinal surgery [5–7]. The reasons for this nonsuperiority could be partially explained by the following two explanations: robotic surgery shares the same operative process as conventional laparoscopic or thoracoscopic procedures; conventional endosurgery is nowadays well optimized, sophisticated, and less invasive, leaving little room for improvement. Therefore, in our opinion, surgical robot should be applied for newly developed techniques and for fields where conventional surgical methods leave technical problems to solve. We believe that our initial experience with this procedure is a breakthrough that will lead to truly minimally invasive surgery for thoracic esophageal cancer.

## Electronic supplementary material

Below is the link to the electronic supplementary material.

Supplementary material 1 (MPG 138692 kb)

## References

[1] Boone J, Schipper ME, Moojen WA, Borel Rinkes IH, Cromheecke GJ, van Hillegersberg R (2009). Robot-assisted thoracoscopic oesophagectomy for cancer. Br J Surg.

[2] Galvanii CA, Gorodner MV, Moser F (2008). Robotically assisted laparoscopic transhiatal esophagectomy. Surg Endosc.

[3] Tandon S, Batchelor A, Bullock R (2001). Peri-operative risk factors for acute lung injury after elective oesophagectomy. Br J Anaesth.

[4] Misthos P, Katsaragakis S, Milingos N (2005). Postresectional pulmonary oxidative stress in lung cancer patients. The role of one-lung ventilation. Eur J Cardiothorac Surg.

[5] Xiong B, Ma L, Zhang C (2012) Robotic versus laparoscopic gastrectomy for gastric cancer: A meta-analysis of short outcomes. Surg Oncol, 10 July [Epub ahead of print] 10.1016/j.suronc.2012.05.004 22789391

[6] Kwak JM, Kim SH, Kim J, Son DN, Baek SJ, Cho JS (2011). Robotic vs laparoscopic resection of rectal cancer: short-term outcomes of a case-control study. Dis Colon Rectum.

[7] Giulianotti PC, Coratti A, Sbrana F, Addeo P, Bianco FM, Buchs NC, Annechiarico M, Benedetti E (2011). Robotic liver surgery: results for 70 resections. Surgery.

[8] Rizk NP, Ishwaran H, Rice TW (2010). Optimum lymphadenectomy for esophageal cancer. Ann Surg.

